# Fermented Polyherbal Formulation Ameliorates the Severity of Acute Multiple Antibiotics-Resistant *Pseudomonas aeruginosa*-Infected Burn Wound in a Rat Burn Model

**DOI:** 10.1155/2024/3601954

**Published:** 2024-05-16

**Authors:** Subhanil Chakraborty, Subhajit Sen, Arghya Das, Ranadhir Chakraborty

**Affiliations:** ^1^Omics Laboratory, Department of Biotechnology, University of North Bengal, Raja Rammohunpur, P.O. NBU, District Darjeeling, Siliguri 734013, West Bengal, India; ^2^DBT-NECAB, Assam Agricultural University, Jorhat 785013, Assam, India; ^3^Department of Microbiology, All India Institute of Medical Sciences, Madurai 625002, Tamil Nadu, India

## Abstract

*Pseudomonas aeruginosa*, a Gram-negative opportunistic bacterium, has emerged as a cause of life-threatening infections in burn wounds. Current therapeutic approaches through wound dressings and systemic medicines are far from satisfactory; resistance to more than two antibiotics shown by pathogens contributes to failures of therapy causing mortality. This animal study was conducted to check the efficacy of one Ayurveda-based fermented polyherbal preparation (AP 01) against multiple antibiotics-resistant (MAR) *P. aeruginosa* HW01*-*infected rat burn wounds. AP-01 was applied on artificially infected burn wound on a rat model infected with MAR *P. aeruginosa* to register the healing effects in terms of reduction in residual wound area percentage, the presence of C-reactive protein in blood, and the presence of viable bacteria colony. Topical application with conventional antibiotics served as a positive control. The polyherbal preparation had reduced the infected residual burn wound area at 40.63% ± 0.69 from the initial burn wound area within two weeks after a single intervention, whereas residual burn wound area remained much higher in the case of animals left untreated and in the case of the animals treated with control drug. Restoration to the normalcy of serum C-reactive protein level was also achieved earlier in the case of polyherbal AP-01-treated groups than in other groups. Fermented formulations using components of AP-01 singly or in different combinations had never been tested earlier for topical application in infected burn wound. The formulation of AP-01 was found superior in terms of the rate of healing and control of infection by MAR *P. aeruginosa* strains in burn wounds in rat models.

## 1. Introduction

*Pseudomonas aeruginosa* is a monoflagellated motile, rod-shaped, non-spore-forming Gram-negative opportunistic pathogen that causes serious infections in plants, animals, and human beings [1]. Notably, inherent broad-spectrum antimicrobial resistance in *P. aeruginosa* isolates has limited the scope of current antibiotics regimen in treating *Pseudomonas* infections and as identified by the Centers for Disease Control and Prevention (CDC), USA; as much as 8% of all healthcare-associated infections were found to be caused by *P. aeruginosa* isolates, among which about 13% are caused by multi-drug-resistant strains attributing more than 400 deaths directly per year in the USA alone [2]. Worldwide, *Pseudomonas* infections affect individuals with a wide array of pathogenic conditions including severe burn wounds. Among the eight categories of antibiotics normally used in clinical practice to treat *P. aeruginosa* infections, multi-drug-resistant or MAR *P. aeruginosa* isolates were identified if it is resistant to more than one therapeutic agent belonging to at least three categories [3].

Ayurveda, the doctrine of ancient Sanskrit scripted resolutions, addressing the health and well-being of human populations and animals had always offered holistic, nature-based curative means in treating wide varieties of diseases, disorders, and pathological conditions. Concocting combinations of extracts from several plant parts according to the Ayurveda formulary to yield a synergistic therapeutic effect against an illness was a common practice among ancient physicians and healers of India. Possible benefits or threats of the Ayurveda-based mono-herbal therapy or polyherbal therapy in treating several disorders were tested through preclinical and clinical research in different types of diseases with very little scientific proceedings toward enriching the potential of therapeutic benefits in designing modern drugs supported by thorough *in vitro* and *in vivo* studies [4]. “Arishtha” preparations are particularly important as these are herbal decoctions made with boiling water where certain plants used are capable of self-fermenting and finally produce stable liquors with the intended therapeutic property [5]. In the current study, we have demonstrated the potential of Ayurveda-based polyherbal preparation (named AP-01; prepared in the laboratory following the Arishtha manufacturing protocols from Ayurveda Pharmacopoeia referred later in the article) was used in treating burn wound infections in a rat burn model. The components of AP-01 singly or in different combinations of two or all mixed have never been tested earlier or applied topically to control burn wound infection whatsoever. The scientific cue to design experiments to control bacteria-infected burn wound arose from the observations that AP-01 was not only able to inhibit swarming and biofilm formation of *P. aeruginosa* but also effective in killing MAR strains of *P. aeruginosa* [4].

The ubiquitous presence of *P. aeruginosa* in water, soil, and different surfaces has helped it easily infect burn wound surfaces and further invasion in the systemic circulation [6]. Colonization and persistence on burn wound surfaces and resistance toward empirical antibiotic therapy have made it emerge as one of the most threatening pathogens in people suffering from burn wounds [7]. Multi-drug-resistant *P. aeruginosa* pathogens are placed on the top priority list of the World Health Organization posing a critical threat to cause morbidity and mortality, and also considered under serious threat level by the CDC because the current arsenal of healthcare professionals in treating *Pseudomonas* infections is falling short day by day [8]. In Europe, 33.9% of *P. aeruginosa* was found to be resistant to at least one of the antimicrobial groups (piperacillin + tazobactam, fluoroquinolones, ceftazidime, aminoglycosides, and carbapenems), according to the report published in 2016 by eCDC or European CDC [3]. The present study was conducted to evaluate the efficacy of fermented polyherbal Ayurveda-based formulation—AP-01 in the treatment of MAR *P. aeruginosa-*infected burn wounds and the prevention of infection spreading through the systemic circulation.

## 2. Materials and Methods

### 2.1. Crude Plant Parts for the Fermented Polyherbal Formulation

Fermented polyherbal formulation (AP-01) was prepared in the laboratory following Ayurveda by boiling dried and cleaned stem barks of *Holarrhena antidysenterica* and *Gmelina arborea* mixed with large-sized dry resins of *Vitis vinifera* in the presence of dried flowers of *Madhuca indica* in 1-liter sterile distilled water. Dried flowers of *Woodfordia fruticosa* [9] were added after the volume of water was lowered to one-quarter of the initial volume through boiling for initiation of fermentation while using Jaggery as a source of sugar [5]. After fermentation was completed in about 45 days, the formulation was duly filtered and kept in sterile dark glass bottles after passing through bacterial filters of 0.2-micron pore size for further use. The method of preparation was per the Ayurvedic Pharmacopoeia of India following references of Sharangdhar Samhita and Bhaisajya Ratnavali for different “Arishtha” formulations [10–12]. Antibacterial activity was tested for each active plant parts of *Holarrhena antidysenterica*, *Gmelina arborea*, *Vitis vinifera,* and *Madhuca indica* for equivalent strength by using *Woodfordia fruticosa* for fermentation and Jaggery as a source of sugar to make Arishtha preparations from individual components of the polyherbal preparation. All plant parts were procured from local sources and identified by a qualified Ayurveda practitioner bearing formal academic degrees for practicing Ayurveda. The safety profile of the polyherbal Arishtha preparation was established from the literature and the physician for both oral and topical routes of administration.

Piperacillin/tazobactam (Lupin Ltd., India) was used as a standard drug in animal studies related to infected burn wound healing by polyherbal preparation AP-01.

### 2.2. Experimental Animals

Thirty-six Sprague Dawley rats of either sex with an average of six weeks old, weighing 120 to 160gm, were obtained from the animal house of the Department of Zoology, University of North Bengal, and distributed randomly into six groups where every group contained equal numbers of male and female animals. Animals were acclimatized for seven days in standard environmental conditions, and a pellet diet of chow and water was provided ad libitum.

### 2.3. Microorganism

The strain *P. aeruginosa* HW01 isolated from the district hospital wastewater [13] was used to induce external infection on the burn wound site in the study of *in vivo* wound healing assay.

### 2.4. Antimicrobial Susceptibility Test

The antibiotic resistance/susceptibility profile of *P. aeruginosa* HW01 was determined as per the guidelines of CLSI (National Committee for Clinical Laboratory Standards Institute) [14]. The Kirby-Bauer disk diffusion method was applied to determine the antibiotic resistance/susceptibility profile of *P. aeruginosa* HW01. Mid-log-phase culture of HW01 (equivalent to 0.4 McFarland standard) was spread on Mueller Hinton agar (MHA) plates by spread plate technique. After spreading the culture evenly over the entire surface of the media, different antibiotic disks (HiMedia, India), such as amikacin-AK (30 *μ*g), aztreonam-AT (30 *μ*g), cefepime-CPM (30 *μ*g), ceftazidime-CAZ (30 *μ*g), ciprofloxacin-CIP (5 *μ*g), doripenem-DOR (10 *μ*g), levofloxacin-LE (5 *μ*g), meropenem-MRP (10 *μ*g), piperacillin/tazobactam-PIT (100/10 *μ*g), ticarcillin-TI (75 *μ*g), and tobramycin-TOB (10 *μ*g), were gently placed on the plates and incubated for 18 h at 37^0^C. After incubation, the zone of inhibition was measured and compared with break points given by CLSI to determine the resistance/susceptibility profile of *P. aeruginosa* HW01 against the abovementioned antibiotics.

### 2.5. Antimicrobial Assay of Polyherbal AP-01 against *P. aeruginosa* HW01

The minimum inhibitory concentration (MIC) of polyherbal preparation AP-01 against the *P. aeruginosa* HW01 was determined by the broth dilution method recommended by the National Committee for Clinical Laboratory Standards Institute (CLSI) [14]. Polyherbal preparation AP-01 was added to sterile 5 ml Mueller Hinton broth in different concentrations from 0 *μ*l/ml to 150 *μ*l/ml with a gradual increment of 10 *μ*l/ml, and then, 1% aliquot of the mid-log-phase culture of *P. aeruginosa* HW01 was inoculated in each tube equally and incubated for 18 h at 37°C. After incubation, growth was measured in a spectrophotometer (SPECTROSTAR Nano—BMG LABTECH) at 600 nm to determine the minimum inhibitory concentration (MIC). All the experiments were done in triplicates and repeated twice. Overnight grown mid-log phase *P. aeruginosa* HW01 culture was inoculated in both the presence of polyherbal AP-01 and piperacillin/tazobactam at MIC dose and without the presence of any drug at 37°C and incubated for six hours to compare the optical density-based growth in both conditions using the spectrophotometer.

### 2.6. Swarming Motility Assay of *P. aeruginosa* HW01

A swarming motility assay of *P. aeruginosa* HW01 was carried out on a soft agar medium following the standard method [15]. Luria–Bertani (LB) broth was supplemented with 0.5% agar and 5.0% glucose to prepare soft agar medium. Liquid polyherbal preparation AP-01 at a volume of 40 *μ*l per ml media was added to a sterile soft agar medium before pouring the test plates. In the case of preparing control plates, sterile distilled water (equivalent to the volume of AP-01 added in the test medium) was added to the sterile soft agar medium before pouring the plates. 10 *μ*l of the overnight grown culture of *P. aeruginosa* HW01 was inoculated in the center of the test and control soft agar plates and incubated at 37°C for at least 18 h in the upright position to compare the diameter of the swarming zone in both test and control plates.

### 2.7. *In Vivo* Wound Healing Assay

*In vivo* wound healing experiment was done by following our previously published research work, by the National Research Council's Guide for the Care and Use of Laboratory Animals [16, 17]. The study was approved by the Institutional Animal Ethics Committee of the University of North Bengal, Siliguri, Distr. Darjeeling, West Bengal, India (Ref. No. IAEC/NBU/2018/04 dated 12.09.2018). Parallel animal studies were done with six Sprague Dawley rats randomly assigned into each group, maintaining the sex ratio of 1 : 1, and one animal was kept per vinyl cage (Tarson, India) to prevent possible aggression, if any, of one animal toward the other of the same sex or opposite. Each rat was considered as the experimental unit. For a total of thirty-six experimental units, there were six groups, and each group had six experimental units. Group 1 (Gr I) was designated for the experiment where burn wound (not infected with *P. aeruginosa* HW01) was given 100 *μ*l sterile distilled water (=untreated). Group 2 (Gr II) was designated for experiment where burn wound infected with *P. aeruginosa* HW01 was given 100 *μ*l sterile distilled water (=untreated). Group 3 (Gr III) was designated for experiment in which topical antibiotics were used to treat burn wounds that were not infected with *P. aeruginosa* HW01. Group 4 (Gr IV) was designated for experiment in which topical antibiotics were used to treat burn wounds infected with *P. aeruginosa* HW01. Group 5 (Gr V) was designated for experiment in which a polyherbal formulation called AP-01 was used to treat burn wounds not infected with *P. aeruginosa* HW01. Group 6 (Gr VI) was designated for experiment in which a polyherbal formulation called AP-01 was used to treat burn wounds infected with *P. aeruginosa* HW01.

There were two positive control groups, Gr III and Gr IV, respectively. There were two study groups, Gr V and Gr VI, respectively. The burn wound area on the rat body surface was measured using “SketchAndCalc” software (https://www.sketchandcalc.com/area-calculator/) and mobile application (freely available from Google Playstore) that works on the principle of digital planimetry. Mobile phone camera was used over the surface of the wound for demarcating the margins of an uneven wound. With the use of “Magic Wand,” multiple points on the edges of irregular surfaces were made to get the perimeter of the burn wound demarcated. The software enabled the calculation of the burn wound area from the two-dimensional perimeter. All the results related to residual wound area percentage, CRP data, and visible signs of wound healings of the parallel groups were compared with each other to draw inferences. The bottom of the cages was lined with rice husk and was kept in an animal enclosure under controlled atmospheric conditions of temperature (25 ± 2°C), humidity (55 ± 5%), and a diurnal variation of 12 hours light and dark period was maintained throughout the study period. Food pellets of standard chow diet (Pranav Agro Pvt. Ltd., India) and machine-filtered tap water (Aquaguard, Eureka Forbes, India) were fed to the animals ad libitum. Any individual animals that showed visible signs of prior illness, fatigue, or fever were excluded from the study. Pregnancy of female rats was also considered as an exclusion criterion. Any animals under 120 gm or over 160 gm were excluded from the study. Overweight rats could possess higher percentage of subcutaneous or epidermal adipose tissues and fat layers causing easy spreading of topical infections to the systemic circulation. Standard randomization prompts using MS Excel were used for randomly including experimental units in each group. Each animal of a group was caged individually in a single marked cage to prevent aggression and licking of the wound surface by other animals to minimize the confounder. The authors SC, SS, and RC were in complete knowledge of the randomization, blinding, and allocation in a particular group for each animal. The outcome of the study was measured in terms of visual inspection of burn wound-healing and reappearance of fur over the wound surface, restoration of the normalcy of serum C-reactive protein values, absence of viable bacterial colonies from the surface of the burn injury, and reduction of residual burn area percentage of up to fifty percent among the positive control group animals. No accidental death of experimental animals occurred or euthanasia was required during the course of the study. Ethical palliative and restorative care was provided to every animal before rehousing the animals in the Department of Zoology for further use.

#### 2.7.1. Burning Procedure

Slight modifications were applied to the method of burning techniques reported by earlier authors to develop an animal burn model [18, 19]. The site for burning was chosen at the dorsal side of rats while keeping equivalent proximity from heads and other internal vital organs. The furs of the rats were shaved from the site on the back after wetting the surface with alcohol swab for providing a local anesthetic and anti-infective effect, covering an equal area from the spinal cord at first with the help of sterile scissors and razor. General anesthesia was performed within a closed-lid wide-mouthed glass vessel using a cotton swab dipped in 2 ml solution of a combination of ether chloroform at a ratio of 1 : 3 v/v for each experimental unit, before placing a piece of fire-proof clothing, carefully over the site chosen for inflicting burn. The site of the intended burn was kept free and visible through a cautiously cut window (at the fire-proof clothing) of one square inch area, which was poured with 0.2 ml of 95% ethanol, while the rest portion of the clothing was held tightly against the rat body surfaces. The poured ethanol was lit up and left to burn, and the flame was extinguished exactly after 15 seconds. Intraperitoneal injection of 0.5 ml physiological saline solution (PSS; 0.85% NaCl) was injected into each animal immediately after the burning episode to revive them from the shock of the burn and anesthesia while they gained consciousness.

#### 2.7.2. Topical Infection on the Burn Wound Site

Animals belonging to three groups were infected by inoculating the wound site with *P. aeruginosa* HW01 hospital wastewater isolate [13]. At first, cell pellets were obtained by centrifuging overnight grown *P. aeruginosa* HW01 cells in Luria–Bertani broth (HiMedia, India) at 4000 revolutions per minute at 4°C, and then, the cell pellet was resuspended in sterile phosphate buffer saline (PBS) after two consecutive washes with PBS to obtain a cell density of 10^9^ bacterial cells per milliliters. An aliquot of 200 *μ*l cell suspension from the same tube was used to infect a single burn zone in each animal under anesthesia. The wound was infected just after the burn was inflicted, while the animals were still under anesthesia. A single layer of sterile nonabsorbent cotton gauze was temporarily placed over the burn area for 10 minutes to aid effective absorption of the inoculum before application of any other treatment [16]. The weights of the rats were taken daily, and the food intake by rats was also noted regularly.

#### 2.7.3. Topical Treatment of Burn-Afflicted Site

All treatments were provided after infecting the wound sites with *P. aeruginosa* HW01 for designated groups and removal of the cotton gauze by using temporary restraints. All liquid topical treatments were given by temporarily immobilizing the animals using physical restraints after ten minutes of burn infliction and externally infecting the burn wound. Animals were already divided into six groups, each group comprising six animals where a male-to-female ratio of 1 : 1 was maintained. Animals of groups I, III, and V were all inflicted with burn, but the burn sites were not inoculated with *P. aeruginosa* HW01 infections, whereas burn wounds of all animals from groups II, IV, and VI were infected with *P. aeruginosa* HW01. Burn wound of Gr I animals (not infected with *P. aeruginosa* HW01) was given 100 *μ*l sterile distilled water (=untreated). The burn wound infected with *P. aeruginosa* HW01 of Gr II animals was given 100 *μ*l sterile distilled water (=untreated). Burn wounds of Gr III animals were treated with 100 *μ*l of piperacillin/tazobactam solution where each milliliter contained 16/4 *μ*g/ml piperacillin/tazobactam (as *P. aeruginosa* HW01 was found susceptible to this combination). Again, 100 *μ*l of piperacillin/tazobactam solution was spread over infected (infected with *P. aeruginosa* HW01) burn wound areas of Gr IV animals. Burn wound sites (not infected with *P. aeruginosa* HW01) of Gr V animals and burn wound sites of Gr VI animals (infected with *P. aeruginosa* HW01) were treated with 100 *μ*l of AP-01. All the thirty-six rats distributed in six different groups undergoing six different treatments were observed for two weeks daily until the disappearance of C-reactive proteins and count of viable *Pseudomonas* colonies from the infected wound site (explained in the later part of this article), and for those two weeks, percentages of residual wound area (PRWA) were measured by the formula: (burn wound area on the day of observation ÷ burn wound area calculated on the day of inflicting burn or zero day) × 100. This calculation took the wound area recorded on the day of inflicting the burn (zero day) as 100% area of burn to reciprocate possible wound healings quantitatively for statistical validation [16].

#### 2.7.4. Qualitative Determination of the Pathogen Load in the Burn Wound Site

Microbiological evaluation was undertaken from the next day of the burning experiment by using “sterile swabs” from the site of infected burn wounds of all animals. The burn wound exudates were collected with the cotton swabs before dipping the entire swab in 1 ml sterile PBS solution. 100 *μ*l aliquot from the swab drained PBS was then aseptically spread over King's Medium A Base (HIMEDIA, M1543-500G) plate. Spread plates in triplicates were evaluated for *P. aeruginosa* HW01 colonies after 24 h of incubation. This evaluation was continued routinely until the disappearance of *P. aeruginosa* HW01 colonies occurred from infected wound sites [16].

#### 2.7.5. Determination of C-Reactive Protein (CRP) Levels Postburn and Infection

Certain volumes of blood samples (15 to 20 *μ*l) of the rats were collected before intervention and after every 48-h intervals from the tail veins, saphenous veins, and dorsal pedal veins in the sterile 0.25 mL PCR tubes for the determination of underlying infections present in the animals. Blood samples were placed vertically for 30–45 min in PCR tubes containing 0.11 M sodium citrate at a ratio of 1 : 10 for anticoagulant to blood to settle down the blood corpuscles. The blood samples were then centrifuged for 5 min at 3600 rpm to separate the serum. Sterile PCR tubes were used for collecting the supernatant serum samples from the blood. The collected serum samples were used to determine qualitative and semiquantitative C-reactive protein (CRP) levels using RHELAX-CRP latex reagent (Tulip Diagnostics (P) Ltd., India). Physiological saline solution was prepared for preparing the testable dilutions of the serum from different specimens. 1 : 2, 1 : 4, 1 : 8, 1 : 16, and 1 : 32 dilutions were made for semiquantitative CRP measurement, and RHELAX-CRP latex reagent was mixed with diluted samples as per the directions provided in the instruction manual. Then, the solution was mixed properly by the mixing stick and the agglutination reaction was observed macroscopically within 2-3 min for proper determination of CRP levels of each animal subjected to the experiment [16].

### 2.8. Statistical Analysis

A total of 288 observations (data of calculated burn wound area) in eight specified days (0, 2, 4, 6, 8, 10, 12, and 14) were transferred to MS Excel format. The area of the burn wound was recorded on specific days of observation per animal (six animals per group) of six treatment groups, resulting in thirty-six observed area per day. The PRWA data on days 2, 4, 6, 8, 10, 12, and 14 of observation were arithmetically calculated using the following formula: (burn wound area on the day of observation ÷ burn wound area calculated on 0 day) × 100. This calculation took the wound area recorded on the day of inflicting the burn (zero day) as 100% area of burn wound. Additionally, all computed values were added to a new column on the current Excel data page. The mean and standard deviation of the six PRWA data points for each treatment were computed using the raw rata and saved to an Excel file on a different sheet. To identify any differences between the six groups, the PRWA data from the 14th day of observation were exported to columns designated for the treatment groups in the one-way ANOVA calculator, which also included Tukey's honest significant difference (HSD) test. It is a post hoc test commonly used to assess the significance of differences between pairs of group means. The null hypothesis for the test states that the means of the tested groups are equal. The *p* value corresponding to the F-statistic of one-way ANOVA was determined to suggest whether 14th-day data on PRWA from one or more treatments were significantly different. This was followed by the Tukey HSD test, Scheffe, Bonferroni, and Holm multiple comparison tests (astatsa.com/OneWay_Anova_with_Tukey HSD/). Bonferroni–Holm method is used to counteract the problem of multiple comparisons. It offers a simple test uniformly more powerful than the Bonferroni correction. These post hoc tests were intended to identify pairs of treatments that are significantly different.

## 3. Results

### 3.1. Antimicrobial Susceptibility Test

As per the antimicrobial susceptibility tests conducted following CLSI guidelines, the hospital wastewater isolate *P. aeruginosa* HW01 was found resistant to ten out of eleven tested antibiotics from different classes of antibiotics (Table 1). It was also found that the isolate was susceptible to the piperacillin/tazobactam combination marginally.

### 3.2. Swarming Motility Assay of *P. aeruginosa* HW01

**Figure 1:**
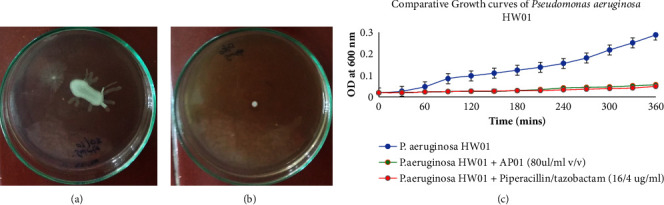
(Color) swarming motility assay of *P. aeruginosa* HW01: (a) control (in the absence of AP-01), (b) in the presence of polyherbal preparation AP-01, and (c) comparative growth curves of *P. aeruginosa* HW01, in the presence of 80 *μ*l/ml (v/v) polyherbal preparation AP-01, 16/4 *μ*g/ml piperacillin/tazobactam, and in the absence of any drug in growth media.

*P. aeruginosa* HW01 formed dendrite-like outward projections from the central point of bacterial inoculation over semisolid media as the branching occurred due to the swarming motility of the bacteria (Figure 1(a)). In the presence of sub-MIC dose of 40 *μ*l per ml polyherbal AP-01 in semisolid media, Figure 1(b) shows that the bacterial cells were able to grow and form an irregular colony at the center but were unable to produce extensive branching or tendrils suggestive of inhibition of swarming motility due to the action of AP-01.

### 3.3. Antimicrobial Assay of Polyherbal AP-01 against *P. aeruginosa* HW01

No visible growth was observed through the spectrophotometric method after 24 hours of incubation at 37°C from the tube containing 82 *μ*l per ml AP-01 and higher drug volumes onward (data not shown). Therefore, the minimum inhibitory concentration of polyherbal AP-01 was determined to be 82 *μ*l per ml against *P. aeruginosa* HW01. Individual components of the polyherbal formulation were assayed separately to check the potentials of the antibacterial effect against *P. aeruginosa* HW01 as shown in Table 2 suggested better efficacy in terms of MIC shown by polyherbal formulation AP-01 than other individual components.  Figure 1(c) depicted no visible growth of *P. aeruginosa* HW01 when it was grown in the presence of a minimum inhibitory concentration of AP-01 and standard antibiotics in Mueller Hinton broth for the first six hours after inoculation with midexponential phase cells. However, in the absence of AP-01, the bacterial cells were found to be growing steadily as shown in the same figure (Figure 1(c)).

### 3.4. *In Vivo* Wound Healing Assay

**Figure 2:**
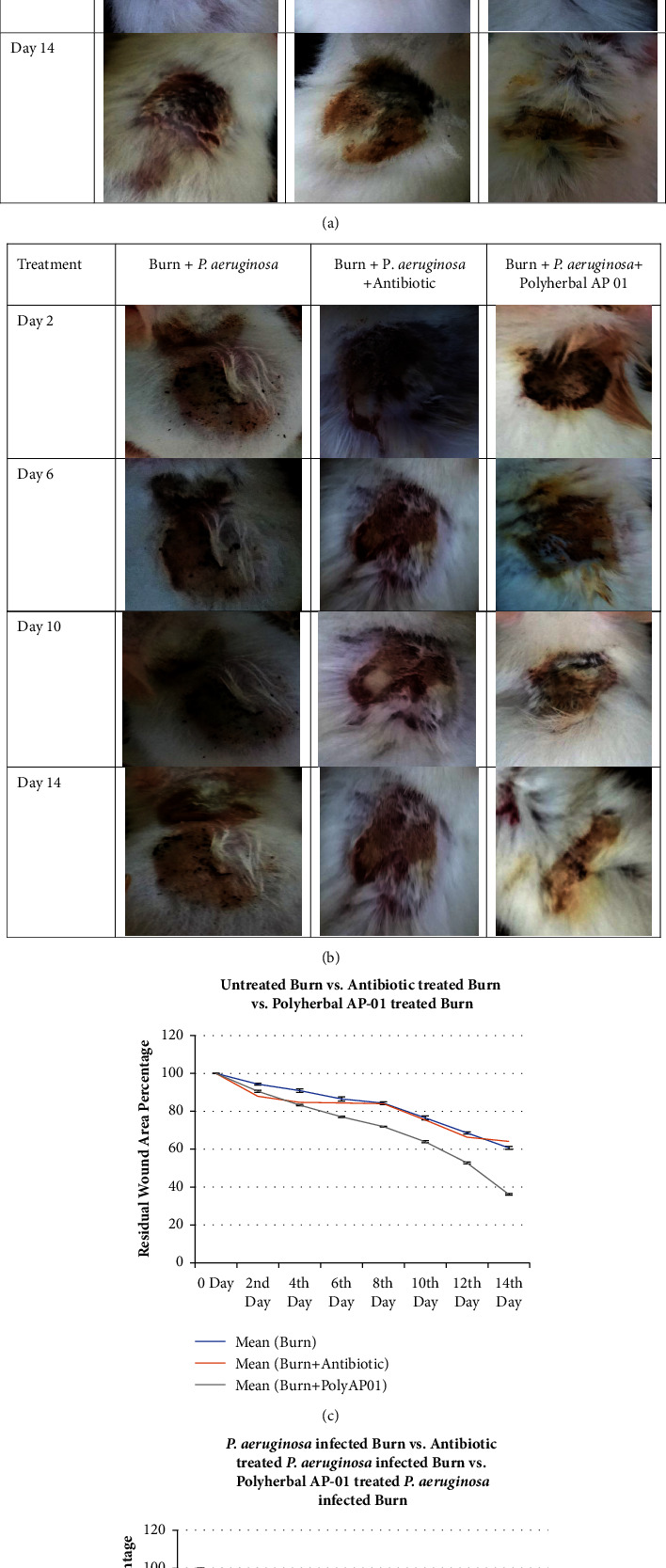
(Color) decrease in residual wound area with progression of burn wound healing: (a) untreated burn (first column), burn treated with antibiotics (second column), and burn treated with polyherbal preparation AP01 (third column); (b) *P. aeruginosa* HW01-infected burn (first column), *P. aeruginosa* HW01-infected burn treated with antibiotic (second column), *P. aeruginosa* HW01-infected burn treated with polyherbal preparation AP-01 (third column), and graphical representation of *in vivo* burn wound healing assay; (c) comparison of residual burn wound area from Day 0 to Day 14 among untreated burn wound, antibiotics-treated burn wound, and polyherbal AP-01-treated burn wound; (d) comparison of residual burn wound area from Day 0 to Day 14 among untreated infected burn wounds, antibiotics-treated infected burn wounds, and polyherbal AP-01-treated infected burn wounds.

Greater burn wound healing and reduction of wound lesion area and reappearance of furs in the burn wound area were also evident in the case of animals treated with polyherbal AP-01 in both infected and noninfected burn groups (Figures 2(a) and 2(b)). Images were taken at the same horizontal and vertical resolution of 220 dpi and focal length of 4 mm. Wound area over the rat dorsal surface was measured using SketchAndCalc® software. An incomplete consistency over the burn wound surface area (considered 100% for each rat) was attained from the flame-burning technique applied in the current study. In animals from groups I, III, and V, where the burn wound was not infected with *P. aeruginosa* HW01, the decrease in residual wound area was found to be maximum in Gr V animals receiving AP-01 treatment and it was determined to be 36.12% ± 0.46 (Figure 2(c)). Animals from groups II, IV, and VI where the burn wound was infected with *P. aeruginosa* HW01, the decrease in residual wound area was also found to be maximum in Gr VI animals receiving AP-01 treatment, and it was determined to be 40.63% ± 0.69 (Figure 2(d)).

It was observed that the burn injury with simultaneous infection of *P. aeruginosa* HW01 caused log-fold increase from the normal level of serum C-reactive protein on Day 2 after burn injury in both treated and untreated animals irrespective of intervention with *P. aeruginosa* infection. The increased CRP level receded faster to normal level with control of inflammation in the animals treated with polyherbal AP-01 (Gr V and Gr VI) within Day 8 when serum CRP became undetectable, whereas increased levels of CRP could be detected in animals of other groups (groups I, II, III, and IV) as shown in Table 3.

The pathogen load for the particular strain of *P. aeruginosa* HW01 gradually reduced to zero number of colonies on the 5th day following burn in the case of AP-01 treated animals of the Gr VI, while detectable numbers of viable bacteria were still present over the infected burn wounds of either the untreated group or the group treated with piperacillin/tazobactam (Table 4).

### 3.5. Statistical Analysis

The F-statistic indicated that there is an overall difference between sample means of PRWA values of the 14th-day observation of 36 rats distributed in six different treatment groups.

The *p* value corresponding to the F-statistic of the one-way ANOVA was found lower than 0.00001, which strongly suggested that one or more pairs of burn wound treatments/groups are significantly different. The result is significant at *p* < 0.05. This was followed by establishing a Tukey test statistic from our sample columns to compare with the critical value of the standardized range distribution. Tukey's HSD procedure facilitated pairwise comparisons with ANOVA data. It allowed determining between which of the various pairs of means—if any of them—there is a significant difference. The result was that the Q value for each pair indicated significant result for all pairs. It is also worth noting that there is no disagreement about whether Tukey's HSD is appropriate because F-ratio score has also yielded significance. Post hoc Tukey HSD test calculator results have revealed a significant difference in the PRWA on the 14th day in the following pairs of treatments. Tukey HSD *p* value for the pairs, Gr-I (burn wound untreated) vs Gr II (burn wound infected with *P. aeruginosa* HW01 remained untreated) or Gr II vs Gr V (a polyherbal formulation called AP-01 was used to treat burn wounds not infected with *P. aeruginosa* HW01) or Gr II vs Gr VI (a polyherbal formulation called AP-01 was used to treat burn wounds infected with *P. aeruginosa* HW01), was 0.0010053. Gr V vs Gr VI had the lowest Tukey HSD Q statistic compared to other treatment pairs (mentioned above) yet produced Tukey HSD *p* value =0.0010053. Scheffe multiple comparison results revealed that the Scheffe *p* value of the treatment pairs I vs II or I vs V or I vs VI or II vs V or II vs VI was 1.1102e-16, while the pair V vs VI has shown Scheffe *p* value =5.519*e* − 10 (Scheffe inference ^*∗∗*^*p* < 0.01). When only pairs relative to I were simultaneously compared, Bonferroni and Holm's results showed that I vs II, I vs V, and I vs VI have yielded highly significant differences with Bonferroni *p* value and Holm *p* value of 0.000*e* + 00. Such a situation is relevant when treatment A is the control and the experimenter is interested only in the differences of treatments relative to the control.

Treatment of burn (with or without *P. aeruginosa* HW01 infections) with antibiotics (piperacillin/tazobactam) produced significant differences, but Bonferroni *p* value and Holm *p* value differed widely: I vs II yielded Bonferroni *p* value and Holm *p* value =0.0000*e* + 00); I vs III (topical antibiotics were used to treat burn wounds that were not infected with *P. aeruginosa* HW01) yielded Bonferroni *p* value = 1.0086*e* − 06 and Holm *p* value = 1.6810*e* − 07; I vs IV (topical antibiotics were used to treat burn wounds infected with *P. aeruginosa* HW01) yielded Bonferroni *p* value = 2.5939*e* − 12 and Holm *p* value = 1.7293*e* − 12; II vs III yielded Bonferroni *p* value =2.6645*e* − 15 and Holm *p* value =2.2204*e* − 13; II vs IV yielded Bonferroni *p* value =1.9363*e* − 11 and Holm *p* value =9.6816*e* − 12; and III vs IV yielded Bonferroni *p* value =2.0811*e* − 07 and Holm *p* value =6.9369*e* − 08. When only pairs relative to I were simultaneously compared, Bonferroni and Holm's results showed that I vs II, I vs III, and I vs IV have yielded highly significant differences with varied Bonferroni *p* values and Holm *p* values.

## 4. Discussion

Burn wounds, like other forms of injury, cause inflammatory and metabolic changes, which trigger a wide range of complications for the patient. After burn injury, the innate immune system counters instantaneously by inducing localized and systemic inflammatory reactions. The innate immune response partakes in triggering the adaptive immune response; conversely, in doing so, it has an undesirable consequence on the burn victim's ability to build up a strong immune reaction to overrunning microorganisms and, as a result, prompts the burn victim to infectious snags. Infection is the predominant reason for morbidity and death in burn-injured populations, with 61% of deaths being caused by infection [20]. Just after the burn incident, the wound surfaces are sterile, but these wounds sooner or later become colonized with microorganisms. Bacteria like Gram-positive Staphylococci, inhabiting sweat glands and hair follicles, can escape burn insult and eventually colonize the burn-affected area in the absence of any topical treatment with antimicrobial agents. Sooner or later, other microbes including Gram-positive, Gram-negative, and yeasts colonize these wounds [21]. For burn patients, *P. aeruginosa* is one such opportunistic pathogen that causes severe, persistent healthcare-associated infections. The majority of failures in burn treatments are linked to inapt primary antibiotic therapy. Heavily burned patients always run the risk of wound infection, and the incidence of infection has a direct correlation with the degree of burn injury. Undoubtedly, topical therapy with the existing antimicrobial agents has considerably decreased the occurrence of invasive *Pseudomonas* burn wound sepsis, but none of the agents sterilize the burn wound. Hence, the chances of the pathogens getting away from the confinement of the burn wound and invading viable tissue increase [22].

Several studies were conducted using herbal drugs (which represent a main part of all traditional systems of medicine) aiming prevention of infection and early wound healing. There are about 1250 Indian medicinal plants [23], of which 46 plants (belonging to 44 genera and 26 families) were known to heal wounds and related injuries, including burn wounds [24]. In one of the studies, burn-injured rats treated with two Ayurvedic formulations, Jatyadi Ghrita and Jatyadi tail, were revealed that the burn wound healing potential of Jatyadi tail was comparable to silver sulfadiazine [25]. Another Ayurvedic preparation is made with *Azadirachta indica A. Juss* leaves, the resin of *Shorea robusta Gaertn. F*, and oil of *Linum usitatissimum* L., called “AtasthadiLep” when applied to the burn wound of a rat, and the percentage wound heal recovery was significantly better than those wounds treated with silver sulfadiazine, but complete eradication of infection was not attained [26]. The present study was planned to elucidate the progress of healing *P. aeruginosa* (MAR strain HW01)-infected burn wound with the fermented polyherbal formulation (AP-01) compared to untreated animals with infected burn wound and treated with antibiotics (piperacillin/tazobactam) to which the strain was found sensitive (Table 1) [4].

The choice and treatment dose of the fermented polyherbal formulation AP-01 over the burning surface was determined from the qualitative swarming-prevention test (Figures 1(a) and 1(b)) and results of the MIC assay (Table 2), respectively. It is evident from this study that the animals suffered burn injury to an extent that confirmed susceptibility to infection with MAR *P. aeruginosa* strain HW01 and enabled us to control burn wound infection or to evaluate the therapeutic efficacy of AP-01. There was a significant difference in the rate of wound healing between untreated and *P. aeruginosa*-infected burn wounds (both Bonferroni *p* value and Holm *p* value are 0.0000*e* + 00). When we looked at PRWA data on the 14^th^ day and then compared the means of two separate groups: Gr IV treatment of *P. aeruginosa*-infected wound with antibiotic, and Gr VI treatment of *P. aeruginosa*-infected wound with polyherbal formulation AP-01 and used *T*-test calculator for two independent means, the t value is −21.01294 and the *p* value is <0.00001. The result is significant at *p* < 0.01 or 0.05, implying that Gr VI (treatment of the infected burn wound with AP-01) was better than Gr IV (antibiotic-treated infected burn wound) supporting Figures 2(c) & 2(d). Again, Tukey HSD *p* value for the pair, Gr II (untreated burn infected with *P. aeruginosa* HW01) vs Gr VI (burn infected with *P. aeruginosa* HW01 and treated with AP01), was 0.0010053 [4]. The increased CRP level ebbed faster to the normal level with control of inflammation in the animals treated with polyherbal AP-01 (Gr V and Gr VI) within Day 8 when serum CRP became undetectable, while for other groups of animals, it was detectable (Table 3). The pathogen load of MAR *P. aeruginosa* HW01 (qualitative assay) was zero in the infected burn wounds on the 5th day following burn in the case of AP-01-treated animals' group (Gr VI), while detectable numbers of viable bacteria were still present over the infected burn wounds of either the untreated group or the group treated with piperacillin/tazobactam (Table 4) [4].

A novel repurposed Ayurveda-based polyherbal formulation as a powerful target-blast-arsenal against notorious MAR pathogens has been developed. Since several different active plant-based components were put together in a single formulation, no bacterium in a sizable population finds an escape route to avoid the deadly assault because the mathematical probability of recruiting multiple mutations simultaneously tends to be zero. Hence, our approach is in stark contrast to the present scenario of the development of resistance within no time against a newly discovered antibiotic. The findings of our animal study revealed success against MAR *P. aeruginosa* infection on rat burn wounds. The same Ayurveda-based polyherbal formulation, AP-01, was also found to inhibit the growth of several MAR gastric pathogens (to be published elsewhere).

## 5. Conclusion

A potent antibacterial formulation derived from a repurposed Ayurvedic polyherbal composition has been produced to combat difficult-to-control MAR infections like burn wound infection. Since numerous active plant-based components were combined into a single formulation, the mathematical likelihood of recruiting multiple mutations simultaneously tends to near zero, meaning that the chances of a bacterium in a large population to find a way out of the lethal attack are much less. Therefore, our strategy contrasts sharply with the current situation where resistance to a newly discovered antibiotic develops quickly. Our animal study's results showed that treating rat burn wounds infected with a MAR strain of *P. aeruginosa* was successful.

As a result, future generations of antimicrobial formulations may employ the Ayurvedic fermented polyherbal composition AP-01 for effective and successful burn wound care.

## Figures and Tables

**Table 1:** Antibiotic resistance profile of *P. aeruginosa* HW01.

Antibiotics	Zone of inhibition (mm)	Zone diameter breakpoints for *P. aeruginosa* (R)	Resistant or sensitive (HW01 isolate)
Amikacin-AK	00	<14	Resistant
Aztreonam-AT	00	<15	Resistant
Cefepime-CPM	04	<8	Resistant
Ceftazidime-CAZ	00	<8	Resistant
Ciprofloxacin-CIP	09	<18	Resistant
Doripenem-DOR	08	<15	Resistant
Levofloxacin-LE	06	<14	Resistant
Meropenem-MRP	08	<15	Resistant
Piperacillin/tazobactam-PIT	22	<14	Sensitive
Ticarcillin-TI	10	<15	Resistant
Tobramycin-TOB	00	<12	Resistant

**Table 2:** Comparative analysis and determined MIC of individual components and the polyherbal AP-01 against *P. aeruginosa* HW01.

Component	Fermentation induced by	Determined minimum inhibitory concentration in *μ*l/ml against *Pseudomonas aeruginosa* (HW01)
*Holarrhena antidysenterica*	*Woodfordia fruticosa*	360 *μ*l/ml
*Gmelina arborea*	*Woodfordia fruticosa*	No inhibition of visible growth
*Vitis vinifera*	*Woodfordia fruticosa*	No inhibition of visible growth
*Madhuca indica*	*Woodfordia fruticosa*	440 *μ*l/ml
*Holarrhena antidysenterica* + *Gmelina arborea* + *Vitis vinifera* *+* *Madhuca indica* (polyherbal AP01)	*Woodfordia fruticosa*	82 *μ*l/ml

**Table 3:** Level of C-reactive protein (CRP) in the blood of different group animals on the second, fourth, sixth, and eighth days following burn and infection with *P. aeruginosa* HW01.

Treatment	Agglutination in different dilutions of the serum						
	1 : 1	1 : 2	1 : 4	1 : 8	1 : 16	1 : 32	mg/dl^#^
*02-day*							
Burn	+	+	+	+	+	+	90−80
Burn + *P. aeruginosa*	+	+	+	+	+	+	90−80
Burn + antibiotic	+	+	+	+	+	−	70−60
Burn + *P. aeruginosa* + antibiotic	+	+	+	+	+	−	70−60
Burn + polyherbal AP-01	+	+	+	+	−	−	60−50
Burn + *P. aeruginosa* + *polyherbal* AP-01	+	+	+	−	−	−	50−40

*04-day*							
Burn	+	+	+	+	+	−	70−60
Burn + *P. aeruginosa*	+	+	+	+	+	−	80−70
Burn + antibiotic	+	+	+	+	−	−	60−50
Burn + *P. aeruginosa* + antibiotic	+	+	+	+	−	−	60−50
Burn + polyherbal AP-01	+	+	+	−	−	−	50−40
Burn + *P. aeruginosa* + *polyherbal* AP-01	+	+	−	−	−	−	40−30

*06-day*							
Burn	+	+	+	+	−	−	60−50
Burn + *P. aeruginosa*	+	+	+	+	+	−	70−60
Burn + antibiotic	+	+	+	+	−	−	60−50
Burn + *P. aeruginosa* + antibiotic	+	+	+	+	−	−	60−50
Burn + polyherbal AP-01	+	+	**−**	−	−	−	40−30
Burn + *P. aeruginosa* + *polyherbal* AP-01	+	+	−	−	−	−	40−30

*08-day*							
Burn	+	+	+	−	−	−	50−40
Burn + *P. aeruginosa*	+	+	+	+	−	−	60−50
Burn + antibiotic	+	+	−	−	−	−	40−30
Burn + *P. aeruginosa* + antibiotic	+	+	+	−	−	−	50−40
Burn + polyherbal AP-01	−	−	−	−	−	−	ND^*∗*^
Burn + *P. aeruginosa* + *polyherbal* AP-01	−	−	−	−	−	−	ND^*∗*^

^*∗*^ND, not detectable (*n* = 6 in each group). ^#^Not a specific quantitatively determined value.

**Table 4:** Presence of *P. aeruginosa* HW 01 viable colonies in seven consecutive days of postburn wound surface in experimental rats.

Treatment	Day 1	Day 2	Day 3	Day 4	Day 5	Day 6	Day 7
Burn	−	−	−	−	−	−	−
Burn + *P. aeruginosa*	+++	+++	+++	++	++	++	+
Burn + antibiotic	−	−	−	−	−	−	−
Burn + *P. aeruginosa* + antibiotic	+++	+++	++	++	++	+	+/−
Burn + polyherbal AP-01	−	−	−	−	−	−	−
Burn + *P. aeruginosa* + *polyherbal* AP-01	+++	++	+	+/−	−	−	−

+++, very high presence [10^4^–10^5^ colony-forming unit (c.f.u.)/ml of swab drained PBS (SDP)]; ++, high presence [10^3^–10^4^ c.f.u./ml SDP]; +, moderate presence 10^2^–10^3^ c.f.u./ml SDP]; +/−, negligible presence [10–10^2^ c.f.u./ml SDP]; −, no presence detected [0–10 c.f.u./ml SDP]. (*n* = 6 in each group).

## Data Availability

The figures, graphs, and tables data used to support the findings of this study are included within the article and also uploaded in the figure's files section.
